# Retinochoroiditis secondary to Rickettsia typhi infection: a case report

**DOI:** 10.1186/s12886-024-03329-5

**Published:** 2024-03-07

**Authors:** Joanne Makhoul, Yael Ben-Arie-Weintrob, Dror Ben Ephraim Noyman

**Affiliations:** 1 Ruth and Bruce Rappaport Faculty of Medicine, Technion-Israel Institute of Technology, Haifa, Israel; 2 Rambam Health Care Campus, Haifa, Israel; 3 Ophthalmology Department, Rambam Health Care Campus, Haifa, Israel

**Keywords:** Retinochoroiditis, Posterior uveitis, Rickettsia typhi, Murine typhus

## Abstract

**Background:**

To report a case of unusual presentation of retinochoroiditis caused by Rickettsia typhi in a patient without prior uveitis.

**Case presentation:**

In this case, we describe a 24-year-old male soldier with no previous eye disease, who was referred to our ophthalmology department due to bilateral retinochoroiditis and vitritis. The patient initially presented with a paracentral scotoma in his right eye persisting for 7 days and scattered dark spots in his left eye for 2 days in June 2023. Preceding these ocular symptoms, he experienced a two-week episode of fever, headaches, night sweats, and rapid weight loss of 10 kg. A transient rash covered his body briefly. His mother had a history of recurrent eye inflammation.

Physical examination revealed bilateral keratic precipitates on the lower corneal periphery, 1 + anterior vitreous cells, small retinal lesions and mild optic discs elevation. Fluorescein angiography indicated mild discs hyperfluorescence, and the clinically visible round punctate lesions on OCT showed inner retinal hyper-reflective lesion with a depth till outer plexiform layer possibly suggestive of a retinitis lesion. Laboratory tests were normal except thrombocytosis, elevated ESR, liver enzymes and ACE levels, with positive Rickettsia typhi serology tests.

Rheumatology and infectious disease consultations ruled out autoimmune diseases, confirming Rickettsia typhi infection. Treatment included systemic doxycycline and prednisone, with improvement of visual acuity, ocular symptoms, OCT abnormalities and resolution of inflammation. Prednisone was discontinued, and after two months, additional improvement was seen clinically, with preserved retinal structures on OCT.

**Conclusion:**

This study explores retinochoroiditis as a rare ocular presentation of Rickettsia typhi, an unusual infection in the Middle East. Previously reported ocular manifestations include conjunctivitis, vitritis, post infectious optic neuropathy and a few cases of uveitis. Ocular symptoms followed systemic illness, highlighting the need for awareness among clinicians. Diagnosis relies on seroconversion, with fluorescein angiography and OCT aiding in assessment. Empiric doxycycline and systemic corticosteroid therapy is recommended. Ocular symptoms resolved in two months. Awareness of these ocular manifestations is essential for timely diagnosis and management. Further research is needed to fully understand this aspect of murine typhus.

## Background

Murine typhus, also known as endemic typhus, is an infectious illness caused by Rickettsia typhi, an obligate intracellular small gram-negative bacterium and a member of the typhus group of rickettsioses. Infection in humans occurs when infected rat fleas deposit their feces into bite wounds [1]. This disease is prevalent in various regions worldwide, particularly in subtropical and tropical areas, notably in port cities and coastal regions where rodents thrive [1, 2]. However, the prevalent organism among the rickettsial agents in the Middle East is Rickettsia conorii, causing Mediterranean spotted fever (MSF) [3]. Therefore, the diagnosis of rickettsia typhi in Israel is rare.

The initial clinical presentation of murine typhus typically manifests after an incubation period lasting from 8 to 16 days. Symptoms include nonspecific clinical signs such as high fever, constitutional symptoms, and a maculopapular rash that can be difficult to discern. Furthermore, it has been linked to inflammatory processes affecting various components of the eye, with a higher prevalence in the retina, optic nerve, and vitreous humor [4]. We report a rare presentation of retinochoroiditis secondary to the unusual infection of Rickettsia typhi in Israel.

## Case presentation

A 24-year-old male soldier with no history of ocular disease or surgery was referred to our ophthalmology department due to bilateral retinochoroiditis and vitritis. In June 2023, he presented with a 7-day history of persistent paracentral scotoma in the right eye and a 2-day history of scattered dark spots in the left eye.

He experienced a two-week episode of a 39 °C fever, headaches, and night sweats preceding the onset of eye symptoms, during which he also lost 10 kg of weight in just 10 days and a mild rash that covered his entire body and resolved within one day. He noted that his mother experienced repeated episodes of eye inflammation with no specific diagnosis. He owned a cat and a dog that were vaccinated and denied any scratches.

The patient denied any other ocular or systemic symptoms, and there was no known familial history of inflammatory or rheumatologic diseases. He denied recent visits to natural reserves such as caves or rivers or contact with other animals.

Upon examination, his best corrected visual acuity was 20/25 bilaterally, and intraocular pressure (IOP) was 12 mmHg bilaterally.

**Fig. 1:**
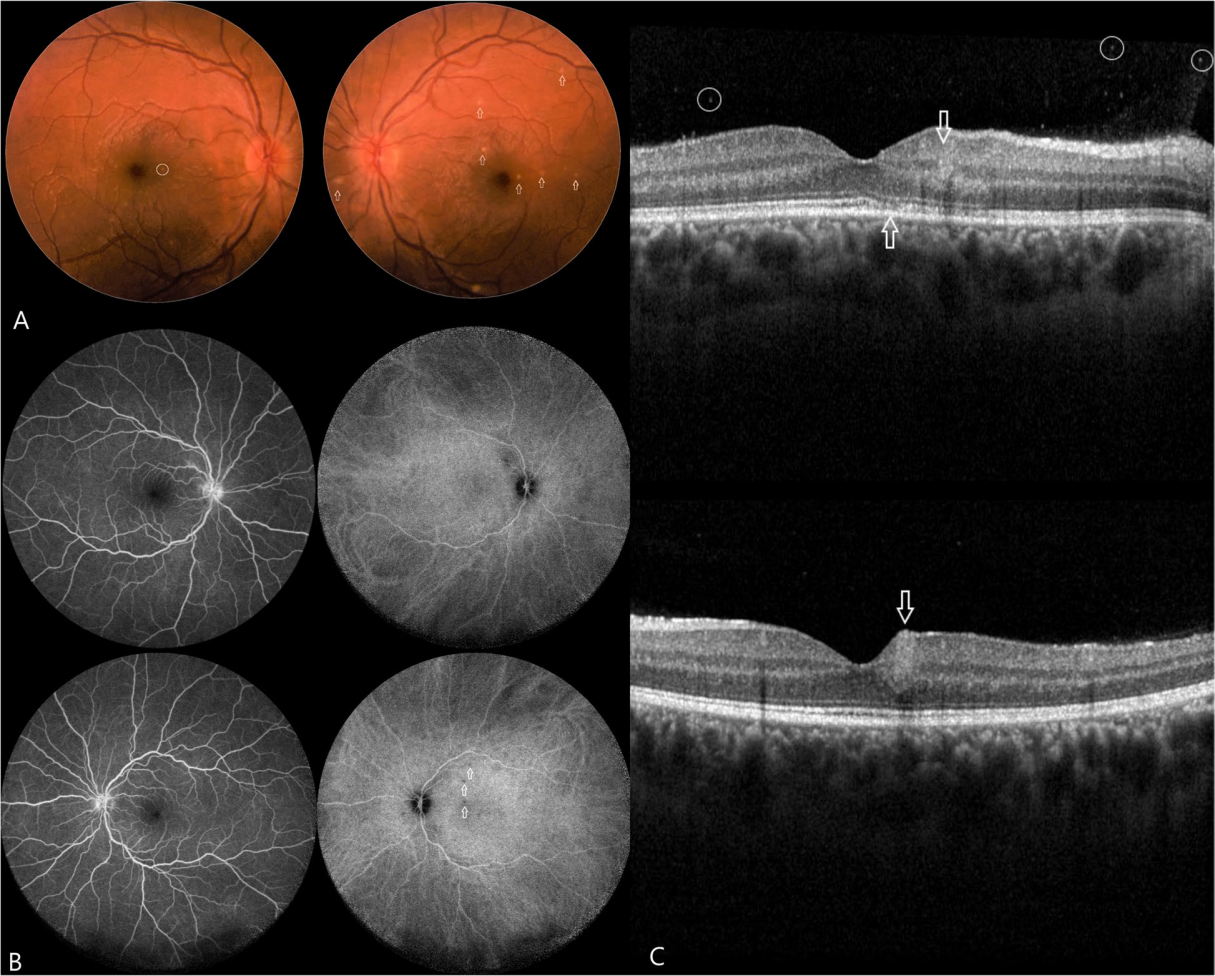
**a** Fundus examination revealed bilateral mild elevation and blurriness of the optic discs nasally. In the right eye, there was a small yellowish small focus consistent with retinitis adjacent to the fovea (*circle)*. Similar findings were found in the left eye in the macular area (*arrows)*. No retinal detachment or signs of inflammation in the periphery were observed. **b** Fluorescein angiography revealed bilateral mild disc hyperfluorescence without vascular leakage. An ICG scan of the right eye showed no focal lesions, while on the left, a few small hypofluorescent foci were observed within the macula and nasally to the disc (*arrows*). **c** OCT of the right eye (*upper image)* revealed hyperreflective retinal areas para-centrally, with irregularities in the photoreceptor layer (*arrows*). Additionally, small hyperreflective foci were observed in the posterior vitreous (*circles*), and the choroid was thickened. In the left eye, a similar pattern can be seen (*white arrow)* but without outer band irregularities

Slit lamp examination revealed a grade 0 reaction with small keratic precipitates (KPs) mainly on the lower corneal periphery and 1 + cells in the anterior vitreous bilaterally. Fundus examination revealed bilateral mild elevation and blurriness of the optic discs nasally (Fig. 1A). In the right eye, there was a small yellowish focus consistent with retinitis adjacent to the fovea. Similar findings were found in the left macula. No retinal detachment or signs of inflammation in the periphery were seen.

Fluorescein angiography (FA) revealed bilateral mild optic disc hyperfluorescence without vascular leakage (Fig. 1B). An ICG scan of the right eye showed no focal lesions, while on the left, a few small hypofluorescent foci were noted within the macula and nasally to the disc.

OCT of the right eye showed a central foveal thickness of 296 μm, with preserved contour and without cystic changes, and choroidal thickening was noted (> 400 μm) (Fig. 1C). Focal retinal hyperreflectivity was noted para-centrally, with irregularities in the photoreceptor layer. Additionally, small hyperreflective foci were observed in the posterior vitreous. In the left eye, the central foveal thickness was 301 μm, exhibiting a similar pattern but without outer band irregularities. The visual fields test yielded normal results.

Laboratory investigations revealed thrombocytosis without leukocytosis, with elevated atypical lymphocyte count. In addition, we found elevated liver enzymes and an elevated ESR of 24 mm/hr. Immunologic tests found elevated ACE levels, mild elevation of immunoglobulins of all types, and normal complement, ANA and ANCA levels. Infectious investigations found negative toxoplasmosis serology and negative RPR and TPHA syphilis test results and positive Rickettsia typhi serology.

The patient underwent a rheumatology consultation that ruled out evidence of spondylitis or lupus. Abdominal ultrasound was performed due to mild impairment in liver function tests and was normal. Chest X-ray, hip joint X-ray, and sacroiliac joint X-ray were all normal. Infectious disease consultation approved the diagnosis of Rickettsia typhi infection. The treatment consisted of doxycycline 100 mg twice daily and prednisone 50 mg daily.

Three weeks later, the patient showed subjective and objective improvement in ocular symptoms. Visual acuity improved to 20/20 bilaterally, with normal IOP. Slit lamp examination revealed clear and unremarkable anterior segments bilaterally. Mild lower vitritis (0.5 + cells) was found in both eyes. Fundus examination revealed mild elevation and slight blurring of the nasal optic disc bilaterally. In the right eye, the macula appeared dry with two small, pinpoint, paracentral well-defined hypopigmented foci and no signs of inflammation in the peripheral retina. In the left eye, a dry macula with a small hypopigmented focus was located nasally to the fovea, and there was no inflammation on the periphery.

The patient was treated with doxycycline for four weeks.

**Fig. 2:**
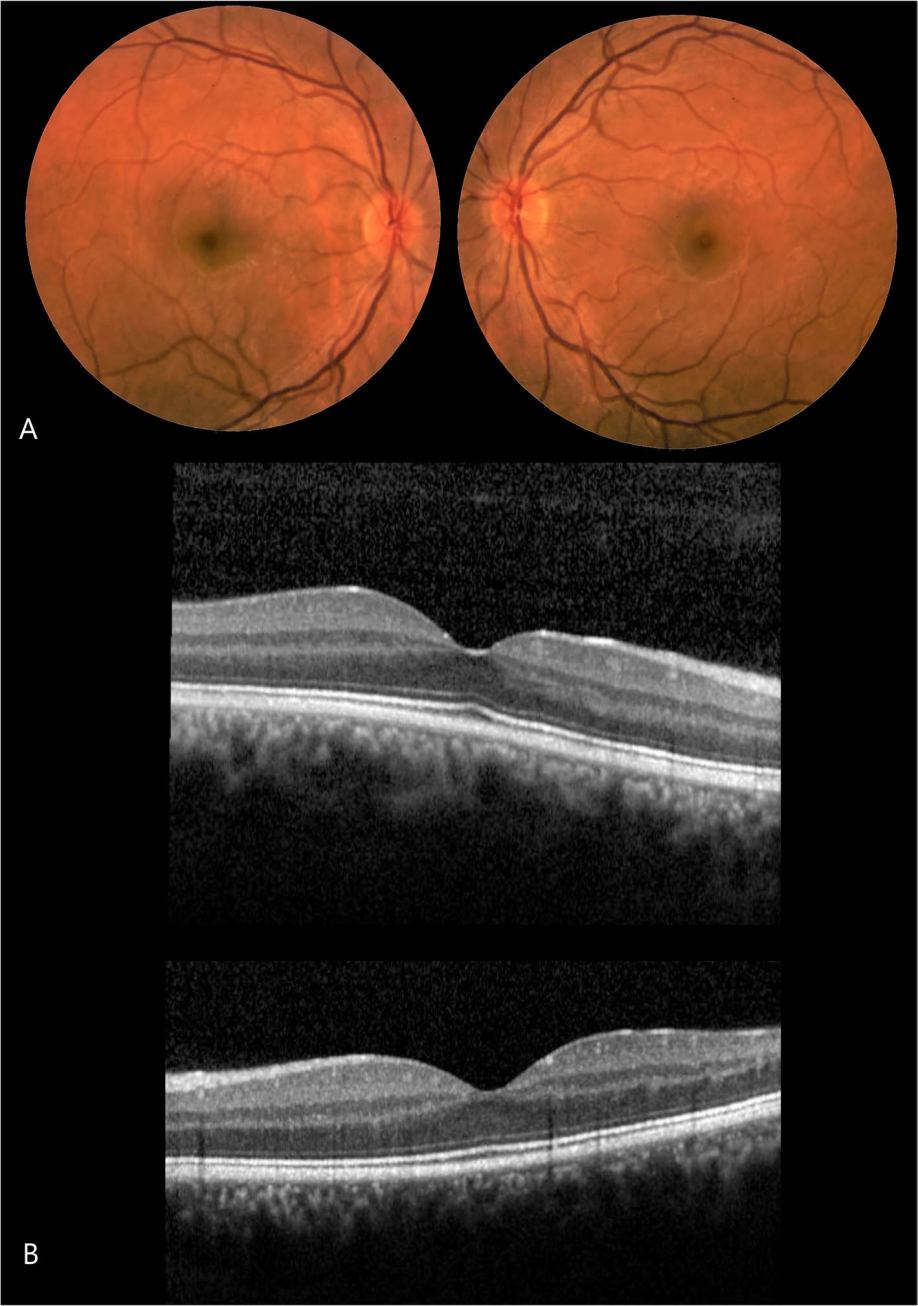
**a** Fundus examination of both eyes revealed few cells in the lower vitreous cavity with unremarkable discs and dry macula **b** OCT of both eyes revealed prominent improvement with preserved contours, and fewer hyperreflective foci with preserved photoreceptor layers

Two months after the initial presentation, the patient showed further ocular improvement. Visual acuity was stable, and fundus examination of both eyes revealed few cells in the lower vitreous cavity with unremarkable discs and dry macula (Fig. 2A). OCT of both eyes revealed prominent improvement with preserved contours and fewer retinal hyperreflective foci with preserved photoreceptor layers (Fig. 2B). He was advised to discontinue prednisone with further follow-up in our ophthalmology clinic.

## Discussion and conclusions

Although murine typhus is found worldwide in warm-climate countries, it is less common in Israel and the Middle East region, where MSF is the prevalent rickettsial disease [3].

While the clinical manifestations of murine typhus have been well documented, its ocular presentations, particularly retinochoroiditis, remain a rare and underexplored facet of the disease.

Previously reported ocular presentations of the disease include conjunctivitis, vitritis, white retinal lesions and vascular leakage, and post-infectious optic neuropathy [5–7]. In addition, two cases of uveitis were reported, with symptoms of scotoma and increased floaters in the setting of persistent fevers and transaminitis, with inner retinal white spots seen on fundus examination [8]. Posterior segment involvement was previously reported mainly in Rickettsia conorii infections, with retinitis in up to 30% of the cases [3, 9].

Here, we report a similar presentation of posterior uveitis, with scotoma and floater and a few retinal white spots, but with the distinct feature of choroidal thickening. Previously reported choroidal involvement included hypofluorescent dots on ICG imaging [7]. Our findings further support the evidence of possible involvement of the choroid in rickettsia typhi infection, with findings of significant choroidal thickening on OCT.

The ocular manifestation occurred immediately after a two-week period of systemic symptoms, including fever, headaches, mild maculopapular rash, night sweats and weight loss, which is similar to the reported range of 6–22 days in prior studies [7]. Signs of retinochoroiditis resolved within two months of the initial onset of symptoms.

Ocular manifestations secondary to Rickettsia typhi can be underdiagnosed due to the long interval between the onset of systemic symptoms and ocular ones and due to the low frequency of systemic symptoms, as the triad of fever, headache, and maculopapular rash presents in less than 33% of reported cases [10].

Fluorescein angiography may be of great yield in the diagnosis and follow-up, as it revealed bilateral mild disc hyperfluorescence. OCT also has a pivotal role, as it revealed hyperreflective areas suggestive of inflammation.

Patients presenting with ocular inflammation, particularly in the context of persistent fever, thrombocytosis, elevated atypical lymphocytes, and elevated transaminase levels, should undergo a comprehensive evaluation for rickettsial infection. This evaluation should encompass a thorough medical history, physical examination, and serologic testing during both the acute and convalescent phases. The gold standard for diagnosis entails seroconversion, defined as a ≥ fourfold increase in serologic IgM and IgG antibodies to Rickettsia typhi during the convalescent phase, detected using immunofluorescence assay (IFA). In certain cases, polymerase chain reaction (PCR) assays of anterior chamber fluid may be considered for pathogen identification; however, such tests are not widely available [11, 12].

In the differential diagnosis, infectious etiologies such as herpes simplex virus (HSV), Epstein‒Barr virus (EBV), syphilis, and tuberculosis should be considered. Noninfectious causes such as Sarcoidosis, Vogt‒Koyanagi‒Harada syndrome, Multiple Evanescent White Dot Syndrome (MEWDS), Punctate Inner Choroidopathy (PIC), and Birdshot Choroidopathy should also be taken into account. Differentiating murine typhus from these conditions can be based on lesion location in the inner retina, along with relevant clinical history and examination findings [7].

The current standard of care involves empiric antibiotic therapy, with doxycycline as the preferred regimen (PO 100 mg BID, or IV for severe infection) for 3–7 days in children or continued for 2–3 days after symptom resolution in adults. In cases where tetracyclines are contraindicated, alternatives such as chloramphenicol and quinolones can be considered. Uveitic manifestations may also necessitate the use of topical and oral steroids [4, 10, 13].

In conclusion, our case report highlights the significance of recognizing retinochoroiditis as a potential ocular manifestation of Rickettsia typhi infection, including the Middle East area and Israel. Ophthalmologists and clinicians should be aware of this rare but distinctive presentation, especially in patients with a history of systemic symptoms and potential exposure to infected fleas. Prompt diagnosis and treatment are pivotal in ensuring favorable outcomes and preventing further ocular complications in individuals affected by this emerging facet of murine typhus. Further research and clinical awareness are warranted to elucidate the full spectrum of ocular manifestations associated with Rickettsia typhi infection and refine diagnostic and management strategies.

## Data Availability

No datasets were generated or analysed during the current study.

## Abbreviations

MSF: Mediterranean spotted fever
IOP: Intraocular pressure
KPs: Keratic precipitates
ICG: Indocyanine green
OCT: Optical coherence tomography
FA: Fluorescein angiography
ESR: Erythrocyte sedimentation rate
ACE: Angiotensin converting enzyme
ANA: Antinuclear antibody
ANCA: Antineutrophil cytoplasmic antibodies
RPR: Rapid plasma reagin
TPHA: The Treponema pallidum haemagglutination
IFA: Immunofluorescence assay
PCR: Polymerase chain reaction
HSV: Herpes simplex virus
EBV: Epstein‒Barr virus
MEWDS: Multiple evanescent white dot syndrome
PIC: Punctate inner choroidopathy
